# *In Utero* Exposure to Environmental Tobacco Smoke Potentiates Adult Responses to Allergen in BALB/c Mice

**DOI:** 10.1289/ehp.9780

**Published:** 2007-01-04

**Authors:** Arthur L. Penn, Rodney L. Rouse, David W. Horohov, Michael T. Kearney, Daniel B. Paulsen, Larry Lomax

**Affiliations:** 1 Department of Comparative Biomedical Sciences, Louisiana State University School of Veterinary Medicine, Baton Rouge, Louisiana, USA; 2 University of Kentucky, College of Agriculture, Lexington, Kentucky, USA; 3 Department of Pathobiological Sciences, Louisiana State University School of Veterinary Medicine, Baton Rouge, Louisiana, USA

**Keywords:** airway hyperresponsiveness, allergens, asthma, environmental tobacco smoke (ETS), *in utero*, tolerance

## Abstract

**Background:**

Fetal stress has been linked to adult atherosclerosis, obesity, and diabetes. Epidemiology studies have associated fetal exposure to maternal smoking and postnatal exposure to environmental tobacco smoke (ETS) with increased asthma risk.

**Objective:**

We tested the hypothesis, in a mouse model of asthma, that *in utero* ETS exposure alters airway function and respiratory immune responses in adults.

**Methods:**

Pregnant Balb/c mice were exposed daily to ETS or HEPA-filtered air (AIR). Offspring inhaled aerosolized ovalbumin (OVA) or saline in weeks 7–8. Regardless of whether they inhaled OVA or saline, mice were sensitized by OVA injections in weeks 11 and 13 followed by OVA aerosol challenge in weeks 14–15. At three time points, we assessed OVA-specific serum immunoglobins, bronchoalveolar lavage cells and cytokines, lung and nasal histopathology, and airway hyperresponsiveness (AHR).

**Results:**

At 6 weeks, we found no significant differences between *in utero* ETS and AIR mice. At 10 weeks, following OVA aerosol, ETS mice displayed greater AHR than AIR mice (α = 0.05), unaccompanied by changes in histopathology, cytokine profile, or antibody levels. At 15 weeks, mice that had inhaled saline in weeks 7–8 developed airway inflammation: eosinophilia (α = 0.05), interleukin-5 (α = 0.05), and AHR (α = 0.05) were greater in ETS mice than in AIR mice. Mice that had inhaled OVA in weeks 7–8 demonstrated no airway inflammation after sensitization and challenge.

**Conclusion:**

*In utero* ETS exposure exacerbates subsequent adult responses to initial allergen exposure.

The incidence of asthma has escalated over the past 20 years. Increasingly, initial asthma episodes are observed early in life (Holt 1998; Weiss 1998). In 2004, nearly 30% of the 30 million diagnosed asthmatics in the United States were < 18 years of age. The highest asthma prevalence rate (148/1,000) exists in 5- to 17-year-olds (American Lung Association 2006). Despite the often early onset of this disease, chronic adult asthmatics comprise the majority of asthma patients.

Stresses in the fetal environment can promote early onset of chronic adult diseases. Initial epidemiologic studies related maternal nutritional deficits to low live birth weights and to premature cardiovascular disease in adult human offspring (reviewed by Barker 2002). In humans, neurologic response to fetal stress has been recently reviewed (Amiel-Tison et al. 2004). In experimental studies, fetal anemia in sheep (Davis et al. 2005) and hypoxia in pregnant rats (Li et al. 2004) have resulted in chronic disease in adult offspring. In pregnant mice, viral infection (Niklasson et al. 2006), pesticide treatment (Cory-Slechta et al. 2005), and environmental tobacco smoke (ETS) exposure (Yang et al. 2004) have also resulted in chronic adult disease in offspring.

Exposure to ETS has been associated with exacerbated asthmatic responses in children (Gilliland et al. 2000; Lindfors et al 1999; Mannino et al. 2001). Altered lung function, increased risk of asthma, and persistent lung function deficits in children have been linked with *in utero* exposure to maternal smoking and/or postnatal exposure to ETS (Gilliland et al. 2000, 2001, 2003; Li et al. 2000; Zlotkowska and Zejda 2005). The synergistic action of tobacco smoke exposure with sensitization to nontobacco allergens may increase the prevalence of allergy and asthma (Oryszczyn et al. 2000). Detailed experimental studies focused on proasthmatic responses resulting from *in utero* ETS exposure combined with postnatal exposure to nontobacco antigens have not been reported.

In rodent models of allergic asthma, ovalbumin (OVA) is a commonly employed antigen for eliciting allergic responses. Sensitization by intraperitoneal (ip) injection, followed by inhalation challenge with OVA elicits expansion of the T helper-2 (Th2) lymphocyte population. Production of Th2 cytokines follows, leading to airway hyperresponsiveness (AHR) and inflammation characterized by eosinophilia and OVA-specific IgE (Zhang et al. 1997).

This sensitization/challenge protocol fails to mimic the typical human experience of aerosol-only sensitization and challenge (Bice et al. 2000; Persson et al. 1997). However, aerosol-only OVA exposure of mice results in little or no OVA-specific serum IgE, and no eosinophilic inflammatory response. This has been attributed to the induction of immunologic tolerance specifically affecting IgE production (Astori et al. 2000; Holt et al. 1981; McMenamin and Holt 1993; Seymour et al. 1998). Interleukin (IL)-10 from regulatory T cells in the lung (Akbari et al. 2001) favors the production of IgG_1_ antibodies and inhibits isotype switching to IgE (Jeannin et al. 1998). This, combined with the induction of T-cell tolerance (Buer et al. 2005), protects most individuals from developing allergic airway diseases to commonly encountered allergens (Tsitoura et al. 2000). Although genetic polymorphisms and underlying regulatory faults in IL-4 and IL-13 production can impair this safety mechanism (Heinzmann et al. 2000; Howard et al. 2001; Izuhara et al. 2000; Seah et al. 2001; Shirakawa et al. 2000;), environmental exposures might also contribute to loss of airway tolerance.

We designed experiments to simulate the respiratory consequences to offspring of pregnant, nonsmoking women exposed daily to ETS. We combined daily ETS exposure of pregnant BALB/c mice with postnatal OVA inhalation to test the hypothesis that *in utero* ETS exposure can alter postnatal respiratory and immune responses to nontobacco allergens.

We posed the following questions. Is *in utero* exposure to ETS sufficient to:

- Compromise respiratory responses in young mice? In adults?
- Promote cytokine production in OVA-exposed mice?
- Influence AHR or lung histopathology following OVA provocation?
- Interfere with establishment of aerosol tolerance to OVA or with production of OVA-specific antibodies?

## Materials and Methods

### Animal protocols

We housed and handled Balb/c mice (Harlan, Indianapolis, IN), according to the NIH *Guide for the Care and Use of Laboratory Animals* (Institute of Laboratory Animal Resources 1996). The Louisiana State University institutional animal care and use committee approved all animal procedures. Animals were treated humanely and with regard to alleviation of suffering. Mice, on a 12-hr light/dark cycle, had food and water *ad libitum* except when in the exposure chambers. We began breeding 8-week-old mice (1 male/2 females) the evening before initiation of ETS exposure. After exposures ended (day 19), we housed pregnant mice in separate cages until weaning of offspring (day 21 after birth).

### ETS exposures

Sidestream smoke, which comprises approximately 90% of ETS, served as a surrogate for ETS (Bowles et al. 2005; Penn and Snyder 1993). A 30-port smoking machine (AMESA Technologies, Geneva, Switzerland) generated smoke from 2R4F filtered research cigarettes (University of Kentucky). We diluted ETS with HEPA-filtered air (AIR) to establish a suspended particle load of 10 mg/m^3^. A MIRAN sapphIRe infrared spectrometer (The Foxboro Co., Foxboro, MA) and a DustTrak particle monitor (STI, St. Paul, MN) continuously monitored carbon monoxide and total suspended particle levels, respectively, in exposure chambers. Gravimetric analyses were performed every 2 hr by weighing 0.45-mm membrane filters (Gelman, Ann Arbor, MI) through which measured amounts of smoke had passed. We exposed 8-week-old mated mice to ETS or AIR (14 air changes/hr, 5 hr/day, 19 consecutive days) in 1.3-m^3^ stainless steel and plexiglass dynamic exposure chambers (71° ± 1.5°F; relative humidity, 53 ± 3%). No offspring were exposed to ETS after birth.

### Aerosol exposures

We exposed mice (7–8 weeks of age) in a 16-compartment, 16.7-L plexiglass inhalation exposure chamber to aerosolized sterile saline or 1% (wt/vol) OVA (Grade V > 98% pure; Sigma Chemical Co., St. Louis, MO) in saline (20 min/day; 10 days). A flow rate of 4 L/min was maintained through an Airlife sidestream high-efficiency nebulizer (Cardinal Health, Dublin, OH).

### OVA sensitization/challenge

We sensitized mice to OVA by ip injections (80 μg OVA in 2.0 mg alum), one each at 11 and 13 weeks of age. OVA challenge included three 20-min inhalation exposures to 1% OVA in saline, every other day at 14 weeks of age, plus a 20-min exposure 1 day before sacrifice at 15 weeks.

### Bronchoalveolar lavage cell and fluid collection

Following euthanasia, we lavaged the right lung of each mouse 4 times with 0.3 mL warm phosphate-buffered saline (PBS) and resuspended the centrifuged bronchoalveolar lavage (BAL) cell pellet in PBS for BAL differential counts (200 cells). Smears were stained with a modified Wright’s stain. The BAL supernatant was stored at −80°C for cytokine analyses.

### Creation of standard sera for isotype-specific ELISAs

We pooled and assayed sera from sensitized/challenged BALB/c mice for OVA-specific IgG_1_, IgG_2a_, and IgE by isotype-specific ELISA at varying dilutions (IgG_1_, 1:10,000; IgG_2a_, 1:100; IgE, 1:10). The pooled sera constituted a standard in subsequent isotype-specific ELISAs on test sera. The isotype-specific optical density (OD) value from the isotype standard was set as one ELISA unit (EU).

### OVA-specific IgG_1_, IgG_2a_, and IgE ELISAs

We used isotype-specific ELISAs to quantitate OVA-specific antibody levels in sera (Seymour et al. 1997) from blood obtained at sacrifice. For IgG_1_ and IgG_2a_, OVA-coated (100 μL/well of 0.1% OVA) 96-well flat bottom plates (Fisher, Pittsburgh, PA) captured OVA-specific antibodies. Horseradish peroxidase (HRP)-conjugated rat anti-mouse IgG_l_ or IgG_2a_ antibodies (BD-Pharmingen, San Diego, CA) marked captured antibody. For OVA-specific IgE detection, 96-well Immulon4 plates (Thermo Electron Corp., Waltham, MA) coated with monoclonal rat anti-mouse IgE (5 μg/mL in PBS, 100 μL/well; BD-Pharmingen) captured serum IgE. Biotinylated-OVA (2.5 mg/mL; Pierce EZ-Link LC; Pierce, Rockford, IL) and HRP-streptavidin (Pierce) marked captured OVA-specific antibody. For all ELISAs, 3,3′,5,5′- tetramethylbenzidine substrate (100 μL/well) detected marked antibodies. We used a standard ELISA reagent kit (OptEIA Reagent Set B; BD-Pharmingen) and converted OD_450_ readings of the samples to EU.

### Histopathologic analysis of lungs

Following BAL collection, we perfused lungs with 0.3 mL freshly prepared 0.02 M periodate–0.1 M lysine–0.25% paraformaldehyde (PLP) fixative in phosphate buffer (pH 7.4), then excised and stored the lungs in PLP for 24–48 hr. We sectioned the cranial, middle, and caudal lobes of the right lung along the plane of each lobar bronchus. Following postfixation in 70% ethanol, we embedded samples in paraffin and dehydrated them directly in graded alcohols to eliminate further exposure to formaldehyde. We stained 3-to 4-μm sections with hematoxylin and eosin. We used a seven-category weighted scoring system (bronchus-associated lymphoid tissue, lymphocytes, plasma cells, eosinophils, neutrophils, mucus metaplasia, and total cellularity) for histopathologic evaluation of lung sections (Bowles et al. 2005). A board-certified veterinary pathologist, blinded to the treatments, evaluated histopathologic samples. A higher score (maximum score = 25) indicates greater tissue responsiveness.

### Histochemical analysis of nasal tissue

We fixed heads in buffered-neutral formalin, then decalcified them for 3 days in 15% formic acid before sectioning (Bahnemann et al. 1995). A pathologist, blinded to the treatments, scored nasal sections for inflammation by cell type and epithelial changes, according to the type of epithelium (squamous, respiratory, and olfactory).

### Cytokine quantitation in BAL fluid

We used a fluorescence-based murine Th1/Th2 cytometric bead array assay (BD-Pharmingen) to analyze aliquots of BAL supernatants on a BD FACSCaliber instrument (BD-Pharmingen). The cytokines included in the assay were IL-2, IL-4, IL-5, tumor necrosis factor-α (TNF-α), and interferon-γ (IFN-γ). Their limits of detection were 5.0, 5.0, 5.0, 2.5, and 6.3 pg/mL, respectively. A single bead assay for IL-13 had limits of detection of 7.3 pg/mL.

### Pulmonary function testing

We assessed AHR in unrestrained mice by whole body plethysmography (Buxco, Troy, NY). Data were expressed as a dimensionless value, Penh, or “enhanced pause” (Hamelmann et al. 1997b). Following acclimation and aerosol saline exposure, we challenged mice with graded doses (1.56–50.0 mg/mL) of nebulized methacholine (Sigma) to assess AHR. We recorded postexposure Penh values over 5 min for each dose.

Although some concern has been expressed regarding the limitations of Penh measurements obtained via unrestrained plethysmography (Lundblad et al. 2002), there are many reports of strong correlations between increases in Penh values and bronchoconstriction demonstrating that Penh serves as a reliable monitor of changes in lung mechanics (Delorme and Moss 2002; Hamelmann et al. 1997a; Singh et al. 2003).

### Statistical analysis

We used the SAS statistical package (version 9.1.3; SAS Institute, Inc., Cary, NC) GLM (general linear model) procedure for all analyses. In addition, where appropriate we conducted an analysis of variance (ANOVA) by sampling time with various end points (BAL cells, histopathology scores, cytokine levels) as the response variables, and treatment, sex, and treatment–sex interaction as effects in the model. We analyzed antibody data by a one-way ANOVA across treatment groups and plethysmograph data in an ANOVA on repeated measures. In considering limits of detection, we ranked cytokine data and carried out a Kruskal-Wallis test (one-way ANOVA) on the ranks. When the overall model indicated significance, we conducted post hoc pair-wise comparisons with Tukey’s HSD (honest significant difference) test for main effects comparisons (Siegel 1956; Steele and Torrie 1980). Pair-wise comparisons of significant interaction effects were conducted with *t*-tests of least square (adjusted) means. In all cases, we considered comparisons significant at α = 0.05.

To test the trend and relationship of one treatment group to another, we paired (blocked) the means of the various response variables and compared the two treatment groups with respect to these paired mean values. The null hypothesis was that the treatment groups were the same based on the paired comparisons. We used the Wilcoxon matched-pairs test, Pearson correlation test, and a regression analysis on the matched groups to examine the potential trend. We considered all analyses significant at α = 0.05 (Neter and Wasserman 1974).

### Exposure and sampling schedule

**Figure 1:**
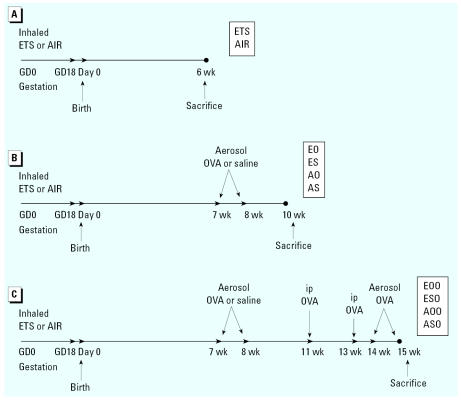
Experimental timeline for prenatal and postnatal treatments and sacrifices showing (*A*) ETS exposure, (*B*) ETS exposure plus OVA inhalation, and (*C*) ETS exposure plus OVA inhalation plus ip OVA challenge. Times are expressed relative to birthdate of offspring. GD, gestation day.

We randomly assigned mated females to gestational ETS- or AIR-exposure groups (a timeline showing the exposure and sampling schedule is presented in Figure 1). At weaning, mice were segregated by sex with no more than four or five mice per cage. We sacrificed groups of *in utero* ETS and AIR mice at 6 weeks of age to determine effects of *in utero* ETS exposure on lung responses of young mice (Figure 1A). Half of the remaining mice inhaled OVA [ETS/OVA (EO), AIR/OVA (AO)] aerosol at 7–8 weeks of age. The other half inhaled saline aerosol [ETS/saline (ES), AIR/saline (AS)]. Each of these groups contained 35–39 mice representing 6–10 litters. We sacrificed mice in each group at 10 weeks of age to determine the effects of *in utero* ETS exposure, plus subsequent OVA inhalation, on lung responses of young adult mice (Figure 1B). We sacrificed the remaining mice, following OVA-sensitization and -challenge, at 15 weeks of age (Figure 1C) to determine the effects of *in utero* ETS exposure on responses to OVA sensitization [ETS/saline/OVA (ESO), AIR/saline/OVA (ASO)] and OVA tolerance [ETS/OVA/OVA (EOO), AIR/OVA/OVA (AOO)]. We analyzed antibody levels, BAL cytology and Th1/Th2 cytokine levels, lung and nasal histopathology, and pulmonary function at all time points.

## Results

Forty to fifty percent of ETS and AIR females had litters. There were no significant differences in delivery rate or litter size between ETS and AIR dams. However, *in utero* ETS offspring were of slightly lower birth weight and experienced a higher mortality rate between birth and 3 weeks of age than did *in utero* AIR offspring (12% vs. 2%; α = 0.05). Thereafter, survival rates were similar, and any inequity in weight or growth rate was lost. In both groups, the male:female offspring ratio was approximately 1:2. At weaning, there were 89 *in utero* ETS offspring from 19 litters and 94 *in utero* AIR offspring from 19 litters.

We detected no upper respiratory tract changes, including evidence of inflammation or congestion, in any treatment groups at any time point during this study, as determined by evaluation of nasal histopathology. We found no differences in any end points between male and female offspring at any time point (data not presented).

### Six-week-old mice

**Figure 2:**
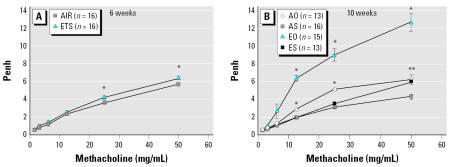
*In utero* ETS exposure has no overall effect in 6-week-old Balb/c mice (*A*) and increases AHR in 10-week-old mice (*B*). Mice received graded doses (1.56–50 mg/mL) of nebulized methacholine, and AHR, expressed as Penh values, was determined by noninvasive, whole-body plethysmography. In (*A*) at the two highest doses, AHR is increased in ETS mice [the asterisk (*) indicates significant difference (*p* ≤ 0.017)]; these points were not sufficient to create a significant difference in overall response curves. In (*B*), *in utero* ETS exposure increased AHR in 10-week-old mice [the asterisk (*) indicates significant difference between all groups; ** indicates significant difference between AS and both AO and ES (*p* < 0.0001)]. Bars represent mean ± SE.

Exposure to ETS *in utero*, without any subsequent treatment, had no overall effect on pulmonary function, although at the two highest methacholine doses a significant AHR increase (*p* ≤ 0.017) was noted in ETS mice (Figure 2A). There were no measurable effects for any of the other end points (data not presented) in 6-week-old mice.

### Ten-week-old mice

At 10 weeks of age, EO and AO mice had consistently differing OVA-specific antibody profiles, IgG_1_ (EO > AO) and IgG_2a_ (AO > EO). These differences however, were not significant for either isotype (data not shown). We detected no OVA-specific IgE in sera of OVA-aerosol exposed mice. As expected, we detected no OVA-specific IgG_1_, IgG_2a_, or IgE in control mice regardless of whether their *in utero* exposure was to ETS (ES) or AIR (AS).

With the exception of TNF-α, all cytokine levels were below limits of detection for all groups at 10 weeks of age. TNF-α was significantly higher (*p* < 0.05; data not shown) in AO mice compared with other groups. Aerosol OVA exposure at 7–8 weeks of age had little overall effect on BAL cell distribution at 10 weeks. As at 6 weeks of age, BAL cells in all groups were mostly mononuclear cells (94–100%). Lung histopathology scores were low, ranging from 6.47 to 7.15. There were no significant differences between any groups in BAL cell distributions or lung histopathology.

Ten-week-old AO mice demonstrated significantly increased AHR compared with AS mice (Figure 2B) at methacholine levels ≥ 12.5 mg/mL. This response was amplified in mice exposed to ETS *in utero*. Ten*-*week-old EO mice exhibited significantly elevated AHR compared with all other groups, at methacholine levels ≥ 6.25 mg/mL. At the highest methacholine concentration (50 mg/mL), the AHR of EO mice was 2 to 4 times greater than that of other groups. Overall response curves were not significantly different between ES and AS mice. However, at the highest methacholine level, ES mice had increased AHR (*p* < 0.0001) relative to AS mice.

### Fifteen-week-old mice

**Figure 3:**
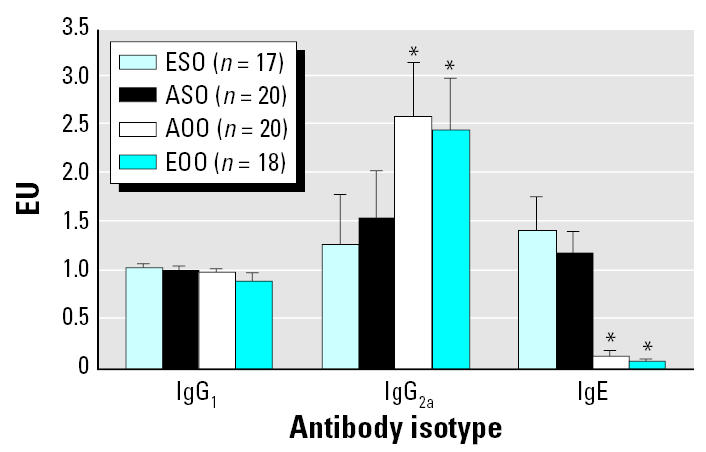
*In utero* ETS exposure has no effect on OVA-specific serum antibody (IgG_1_, IgG_2a_, IgE) levels in 15-week-old mice. Bars represent mean ± SE. *Significantly different from both ASO and ESO (α = 0.05).

We analyzed all remaining mice for the effects of *in utero* ETS exposure on responses to the OVA sensitization and challenge. There were no significant differences in OVA-specific IgG_1_, IgG_2a_, and IgE levels between groups of mice exposed to OVA for the first time (ASO and ESO) or between groups previously exposed to OVA (AOO and EOO; Figure 3). In the former, there were significantly lower levels of OVA-specific IgG_2a_ and significantly higher OVA-specific IgE levels than in the latter.

All measured cytokines were higher in ESO mice than in ASO mice, although not significantly (Table 1). IL-4, IFN-γ, and TNF-α were significantly lower in EOO mice compared with AOO mice. Levels of IL-4, IL-5, IL-13, and TNF-α were significantly lower in EOO mice than in ESO mice. IL-13 and IL-5 were consistently higher in mice exposed to OVA for the first time (ESO and ASO) than in mice previously exposed to OVA (EOO and AOO). Levels of IL-2 did not differ between any groups.

**Figure 4:**
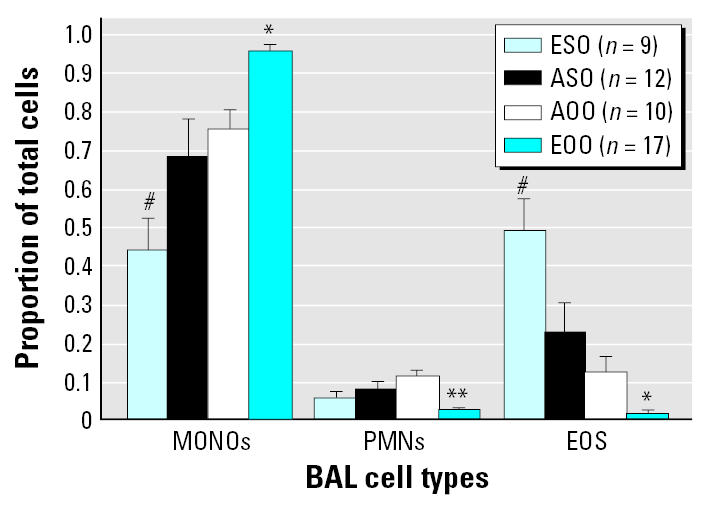
*In utero* ETS exposure affects BAL cell differentials in 15-week-old mice. Abbreviations: EOS, eosinophil; MONO, monocyte; PMN, polymorphonuclear leukocyte. A 200-cell differential count was performed on BAL cells from each mouse in every group. Bars represent mean ± SE. *Significant difference between all groups, **significant difference between EOO and both AOO and ASO, and #significant difference between ESO and both AOO and EOO; α = 0.05.

Eosinophil levels in BAL fluid were significantly higher in ESO and ASO mice than in AOO and EOO mice (Figure 4). Eosinophil and polymorphonuclear leukocyte levels were significantly lower in EOO compared with AOO mice. Mononuclear cells accounted for > 96% of the BAL fluid cells in EOO mice. No BAL cell differences were found between AOO and ASO mice.

**Figure 5:**
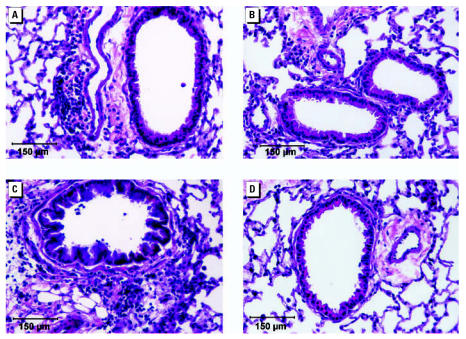
Photomicrographs from the median histopathology score mouse of each group: (*A*) ASO, (*B*) AOO, (*C*) ESO, and (*D*) EOO. Inflammatory responses are most pronounced in ESO mice and least pronounced in EOO mice at 15 weeks of age. Bars = 150 μm.

Increased lung inflammation was evident in both ESO and ASO mice (Figure 5). Lung inflammation in mice previously exposed to OVA (EOO and AOO) was reduced compared with that in mice exposed to OVA for the first time (ESO and ASO). Again, the largest difference was between ESO and EOO mice. There were no differences between EOO and AOO or between ESO and ASO.

**Figure 6:**
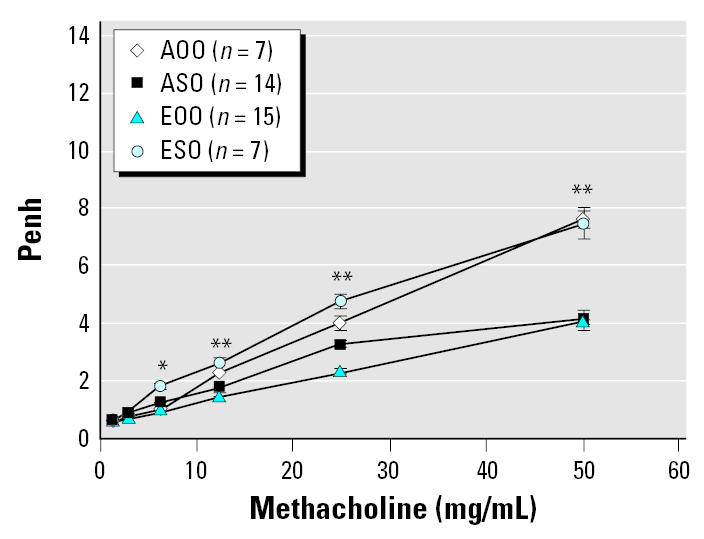
*In utero* ETS exposure alters AHR in 15-week-old Balb/c mice transiently exposed to methacholine 1 day after OVA challenge. Bars represent mean ± SE. *Significant difference between ESO and all other groups (*p* < 0.0001). **Significant difference between ESO and both ASO and EOO (*p* ≤ 0.013) and a significant difference between EOO and both AOO and ESO (*p* ≤ 0.023). EOO differed significantly from ASO only at 25 mg/mL methacholine; this one point did not create a significant difference in overall response curves.

There was increased AHR, as expected, at methacholine levels of 12.5–50 mg/mL in mice exposed to OVA for the first time (ESO and ASO; Figure 6) compared with mice that had previous OVA-exposure (EOO and AOO). However, AHR in ESO mice was also significantly elevated compared with ASO mice. Overall, the most striking differences in AHR were between the responsive ESO mice and the relatively nonresponsive EOO mice. The EOO mice also exhibited significantly attenuated AHR compared with AOO mice.

As we analyzed our data, we noted what appeared to be trends in overall treatment group responses. To further investigate these trends, we identified 11 end points that have a direct relationship to airway inflammation and/or Th2 responses (i.e., they all increase as inflammation and/or Th2 responses increase). We compared paired group means for these end point measures in both *in utero* ETS and *in utero* AIR mice using specific tests for trend (Table 2). Analyses confirmed significant trends in the relationship between ETS and AIR mice.

## Discussion

We determined no differences between sexes in responses of mice exposed *in utero* to ETS and subsequently to OVA. There are no equivalent experimental asthma studies with which to compare these responses directly, because the ETS/OVA exposure schedule we followed has not been reported previously in a mouse model of asthma. There are, however, reports of differences between males and females with regard to asthma end points after postnatal ETS and OVA exposures in Balb/c mice (Melgert et al. 2005; Seymour et al. 2002). Also, differences between sexes have been observed in humans with regard to asthma end points. More adult females than males have asthma, although this relationship is reversed in children (American Lung Association 2006). Mortality due to asthma is higher among human females than among males.

Our studies were designed to address three distinct, but related issues in a mouse model of asthma: *a*) whether there are any long-term, asthma-related consequences arising from *in utero* exposure to ETS, in the absence of any subsequent lung provocation; *b*) what effects, if any, *in utero* ETS exposure has on adult responses to sensitization and challenge with an otherwise innocuous antigen (OVA); and *c*) whether *in utero* exposure to ETS affects establishment of aerosol tolerance.

Human epidemiologic studies suggest that *in utero* ETS exposure may be sufficient to increase the incidence and severity of asthma. Temporal associations between tobacco smoke exposures of fetuses/young children and subsequent altered lung responses have been investigated. The association between smoke exposure and subsequent decreases in forced expiratory volume (a measure of lung function) was stronger when exposure was prenatal (Cunningham et al. 1994). The asthma risk for children exposed *in utero* via maternal smoking was > 2 times that for children receiving postnatal ETS exposure (Cunningham et al. 1996).

Both postnatal ETS exposures and *in utero* smoke exposures have been linked with decreased lung function in school-age children (Gilliland et al. 2000), especially in those already diagnosed with asthma (Li et al. 2000). Furthermore, *in utero* exposure to maternal smoking, without subsequent ETS exposure, was associated with increased asthma and wheezing in young children. Postnatal ETS exposure was, however, associated with increased wheezing but not with increased asthma prevalence (Gilliland et al. 2001). In contrast, in the absence of known allergen exposure, postnatal exposure to ETS was more strongly correlated with increased AHR and wheezing than was ETS exposure during pregnancy (Lindfors et al. 1999).

In a 2001 review of the literature, Lodrup Carlsen and Carlsen (2001) concluded that

[B]oth *in-utero* and, to some degree, passive (environmental) tobacco smoke (ETS) exposure adversely affect pulmonary function, and predispose to asthma symptoms.

A questionnaire-based survey of parents of ≥ 4,000 children revealed that *in utero* exposure to maternal smoking was a strong risk factor for wheezing and physician-diagnosed asthma in young children (Lannero et al. 2006). Neonates of nonsmoking mothers exposed to ETS during pregnancy had higher serum levels of cotinine, (a major nicotine metabolite) than their mothers (Perera et al. 2004). This tendency to bioaccumulate smoke components could cause *in utero* ETS-exposed children to be particularly susceptible to respiratory problems.

Rodent studies have confirmed that *in utero* exposure to ETS can be critical to subsequent respiratory and immune system responses. Rats exposed to sidestream smoke for the first 100 days of life displayed no altered lung function or reactivity to methacholine (Joad et al. 1993). In contrast, female rats exposed continually to sidestream smoke *in utero* from gestation day (GD) 3 through week 10 after their birth, demonstrated a 24% decrease in lung dynamic compliance and a 20-fold increase in methacholine reactivity compared with rats exposed to smoke only during gestation or only after birth (Joad et al. 1995). The timing effect was further refined when rats were exposed to filtered air or sidestream smoke (1 mg/m^3^) *in utero* from GD3 through day 21 after their birth (Joad et al. 1999). At 8 weeks of age, they displayed significant increases in AHR and pulmonary artery pressure and decreases in dynamic compliance compared with controls. In a recent report, Ng et al. (2006) associated increased tumor incidence and growth with *in utero* exposures to mainstream smoke. Offspring of B6C3F1 mice exposed *in utero* to smoke from GD4 to birth, were injected with lymphoma cells at 5 and 10 weeks of age. Males exhibited a > 2-fold increase in tumor incidence and more rapid tumor growth relative to controls. This was accompanied by decreased cytotoxic T-lymphocyte activity without changes in natural killer cell activity, cytokine levels, lymphoid organ histology, or immune cell subpopulations.

Our results from 6-week-old mice indicate that *in utero* ETS exposure without further lung challenge has no observable effect on pulmonary function or histology. The only significant finding at this age is elevated AHR in ETS mice at the two highest methacholine challenge doses. These points are insufficient to produce a significant difference in the total response curve. However, they may reflect an ETS-induced AHR sensitivity that becomes more apparent with additional lung stress or challenge.

At 10 weeks of age, groups of both ETS and AIR mice were exposed to aerosol OVA in the absence of any adjuvant, including lipopolysaccharide, to produce mice that were tolerant to airway inflammation by OVA sensitization and challenge. The antibody profiles of these animals were as expected. There were significant OVA-specific antibody responses (IgG_1_ and IgG_2a_) indicating recognition and allergen-specific activation, but no indication of significant effector response (eosinophilia, mucus hyperplasia, cytokine elevation, or OVA-specific IgE). These findings are consistent with prior studies (Holt et al. 1981; Swirski et al. 2002). As previously reported (Renz et al. 1992), AHR was significantly elevated in OVA mice relative to non-OVA mice. However, the AHR of EO mice was increased significantly relative to AO mice, demonstrating an ETS-dependent exacerbation of AHR to initial allergen exposure. This exacerbation was independent of classical inflammatory or Th2 end points, an occurrence that has been previously described (Barrett et al. 2002). As was the case in 6-week-old ETS mice, 10-week-old ES mice demonstrated a significant increase in AHR compared with AS mice at the highest methacholine dose. Again, this was not sufficient to create a significant difference in the total response curve.

Without subsequent provocation, *in utero* ETS does not alter basic lung histology as evidenced by the lack of significant differences between ETS and AIR mice (6 weeks) and ES and AS mice (10 weeks). However, the AHR increase in ETS groups at our maximum methacholine challenge suggests that AHR has been enhanced by ETS exposure. This is supported by subsequent increases in AHR following respiratory challenge with OVA (EO versus AO).

At 15 weeks of age, after OVA sensitization and challenge, ESO and ASO mice were the most responsive, as reflected by increased Th2 and inflammatory cytokines, respiratory eosinophilia, AHR, and OVA-specific IgE. The EOO and AOO mice were OVA-tolerant with regard to cytokines, IgE, and airway inflammatory markers, especially when compared with ESO and ASO mice. Overall, EOO mice were significantly less responsive than AOO mice in AHR, inflammatory cytokines, and BAL eosinophils and neutrophils. ASO mice exhibited classical markers of airway inflammation but no increased AHR. AOO mice had no signs of airway inflammation but had increased AHR. These results, combined with those of 10-week-old EO mice, support the conclusion that adaptive and innate immune factors may be sufficient, but are not essential, for enhanced AHR in this murine asthma model. In addition, the results at 10 and 15 weeks demonstrate that *in utero* ETS exposure aggravates AHR following initial exposure to OVA, regardless of the route of OVA administration.

Pair-wise comparisons of group means at 15 weeks (Table 2) revealed significant differences for some responses of EOO compared with AOO mice (e.g., BAL differentials, cytokines, AHR) and some responses of ESO versus ASO (e.g., BAL differentials and AHR). However, in all cases, responses were most attenuated for EOO mice and most pronounced for ESO mice.

The Wilcoxon test on paired sign data confirmed that responses in EOO mice were uniformly lower (*p* = 0.001) than in AOO mice, indicative of a significant trend. This was further strengthened by a Pearson correlation value of 0.96 and a ranked regression yielding an adjusted *R*^2^ of 0.91. This analysis supports the conclusion that exposure to ETS *in utero* suppressed responses to OVA sensitization/challenge in mice previously exposed to OVA. The Wilcoxon test on paired sign data also demonstrated that responses in ESO mice were consistently higher (*p* = 0.001) across all data compared with responses in ASO mice. This trend was further supported by a Pearson correlation value of 0.99 and a ranked regression yielding an adjusted *R* ^2^ of 0.98. These results support the conclusion that *in utero* ETS exposure exacerbates subsequent adult responses to initial allergen exposure.

At 15 weeks of age, ESO mice had enhanced initial responses to OVA sensitization and challenge, whereas EOO mice had dampened responses upon reexposure to OVA. The reason for this response divergence is not intuitively obvious. The end point measured in ESO is, indeed, an initial response to OVA. However, because of the “memory” inherent to adaptive immune responses, the measurement in EOO mice at 15 weeks is a secondary response reflecting the initial response to aerosol OVA at 10 weeks. Although that exposure was without adjuvant, establishment of tolerance to OVA is expected. In the case of EOO, the decreased responsiveness at 15 weeks implies increased resistance to OVA sensitization and challenge. Our data do not support a role for *in utero* ETS in determining the type (sensitization or tolerance) of immune response to allergen. However, *in utero* ETS appears to significantly influence the magnitude of response, which may increase sensitivity to allergen or severity of disease.

Adult exposure to diesel exhaust particles, another major environmental combustion product, has been linked with increased incidence of allergies and asthma (Davies et al. 1998; Peterson and Saxon 1996), and rodent studies have confirmed that diesel exhaust particles promote allergic and asthmatic immune responses (Nel et al. 1998; Pandya et al. 2002). These adult studies suggest that environmental exposures do not cause immune deviation but have an adjuvant-like effect on immune response to allergen. We demonstrate that, in addition to aggravating AHR, *in utero* ETS exposure promotes initial immune responses to allergen sensitization and challenge in adults (ESO vs. ASO).

Residual oil fly ash (ROFA) overcomes aerosol tolerance in young mice if the ROFA and OVA exposures are simultaneous (Hamada et al. 2000). Similarly, concurrent administration of inhaled OVA and Th2-adjuvant prevented establishment of OVA-specific IgE tolerance (Hurst et al. 2001). Once tolerance had been established, however, it could not be completely overcome by later simultaneous administration of OVA and the Th2 response-provoking agents. The latter finding is supported by our results, where tolerance resulting from OVA aerosol at 7–8 weeks of age persisted at least 8 weeks and was not overcome by *in utero* ETS exposure. In fact, the dampened responses of adult EOO mice suggest increased resistance to sensitization and challenge. These results are consistent with recent reports that smoking-associated AHR increases can occur independent of changes in levels of Th2 cytokines and IgE (Robbins et al. 2005), and that smoking-allergen interactions may be allergen-specific (Jarvis et al. 1999).

Although the mechanism underlying the ETS-mediated responses to initial OVA exposure has not been identified, preliminary results point to the involvement of arginase-1 (ARG1) in this process. Up-regulation of ARG1 has been identified as an important step in the pathophysiology of asthma (Erdely et al. 2006; Zimmerman et al. 2003; Zimmermann and Rothenberg 2006). Nitric oxide production, which is vital for relaxation of airway smooth muscle cells, depends critically on the presence of adequate levels of arginine. Up-regulation of ARG1 leads to increased catabolism of arginine, decreased NO production, and subsequently increased AHR. Consistent with this, polymerase chain reaction analysis revealed that ARG1 was up-regulated in lungs of ESO versus EOO mice at 15 weeks (Penn et al., unpublished data).

The results of this 15-week study indicate that *in utero* ETS suppresses adult responses to repeated exposures of the same antigen(EOO), but it also aggravates adult responses to initial antigen exposure (EO at 10 weeks of age and ESO at 15 weeks). If this heightened sensitivity extends beyond OVA to unrelated antigens, as seems likely, mice exposed *in utero* to ETS and then to other antigens as adults (e.g., *Aspergillus*, cockroach antigen, bacterial lipopolysaccharide, respiratory viruses) would have exaggerated responses compared with mice not exposed *in utero* to ETS.

## Conclusion

Four main conclusions emerge from this investigation:

- *In utero* ETS exposure alone does not alter respiratory structure or function in healthy mice.
- *In utero* ETS exposure exacerbates initial adult responses to allergen as demonstrated by the EO versus AO at 10 weeks (AHR) and the entire range of ESO versus ASO responses at 15 weeks. This effect is independent of whether OVA is first encountered as a tolerizing aerosol (EO at 10 weeks) or as part of a sensitization and challenge protocol (ESO at 15 weeks).
- In this model, increased AHR upon initial allergen exposure is not necessarily coupled with changes in histopathology, cytokine profile, or antibody levels (compare responses after OVA exposure at 10 vs. 15 weeks) but is related to *in utero* ETS exposure. AHR is sometimes the only significant finding. Coincidentally, in human asthmatic episodes, AHR is the preliminary clinical response and often the only initial finding.
- Finally, OVA tolerance is not overcome by *in utero* ETS exposure.

## Figures and Tables

**Table 1:** Ranked cytokine measurements in BAL fluid of 15-week-old mice.

	IL-13	IL-4	IL-5	TNF-α	IFN-γ	IL-2
AOO (*n* = 21)	1.05*	42.76**	7.24*	31.45**	37.21**	9.07
EOO (*n* = 18)	1.00*	20.14*	1.17*	11.56*	18.36*	2.83
ESO (*n* = 14)	4.57**	48.82**	26.43**	31.96**	35.61	7.54
ASO (*n* = 19)	3.18	39.79**	18.37**	21.53	35.40	4.00

Data were ranked to account for values below the limit of detection. Ranked data were analyzed using a Kruskal-Wallis test (one-way ANOVA). When significant, Tukey’s HSD test was used for post hoc comparisons of effects. For each cytokine, values marked with one asterisk (*) are significantly different from those with ** (α = 0.05); values without an asterisk are not significantly different from any other value.

**Table 2:** Paired-means of treatment groups used for trend analysis.

	BAL cytokines^a^				BAL Eos (%)	Methacholine (mg/mL)^b^			Path^c^ score	Antibodies^d^	
	IL-13	IL-4	IL-5	TNF-α		12.5	25	50		IgG1	IgE
ESO versus ASO											
ESO	4.46	48.82	26.43	31.96	50.0	2.86	4.90	7.68	19.3	1.28	0.20
ASO	3.18	39.79	18.37	21.53	23.0	1.74	3.20	4.04	17.7	0.46	0.18
EOO versus AOO											
EOO	1.000	20.14	1.17	11.56	1.5	1.51	2.31	4.05	10.4	0.90	0.08
AOO	2.300	42.76	7.24	31.45	11.9	2.28	4.00	7.62	12.8	1.00	0.13

Eos, eosinophils.

a BAL cytokines are ranked values.

b AHR is expressed as an index value, Penh, at three methacholine concentrations.

c Cumulative histopathologic index value.

d Expressed in EU.
